# Targeting DYRK1B suppresses the proliferation and migration of liposarcoma cells

**DOI:** 10.18632/oncotarget.22743

**Published:** 2017-11-28

**Authors:** Hua Chen, Jacson Shen, Edwin Choy, Francis J. Hornicek, Aijun Shan, Zhenfeng Duan

**Affiliations:** ^1^ Department of Emergency Surgery, ShenZhen People’s Hospital, 2nd Clinical Medical College of Jinan University, Shenzhen, Guangdong Province, China, 518020; ^2^ Sarcoma Biology Laboratory, Department of Orthopaedic Surgery, Massachusetts General Hospital and Harvard Medical School, Boston, Massachusetts 02114, USA; ^3^ Department of Orthopaedic Surgery, David Geffen School of Medicine at UCLA, Los Angeles, CA 90095-6902, USA

**Keywords:** DYRK1B, liposarcoma, proliferation, migration, apoptosis

## Abstract

Liposarcoma is a common subtype of soft tissue sarcoma and accounts for 20% of all sarcomas. Conventional chemotherapeutic agents have limited efficacy in liposarcoma patients. Expression and activation of serine/threonine-protein kinase dual-specificity tyrosine-(Y)-phosphorylation regulated kinase 1B (DYRK1B) is associated with growth and survival of many types of cancer cells. However, the role of DYRK1B in liposarcoma remains unknown. In this study, we investigated the functional and therapeutic relevance of DYRK1B in liposarcoma. Tissue microarray and immunohistochemistry analysis showed that higher expression levels of DYRK1B correlated with a worse prognosis. RNA interference-mediated knockdown of DYRK1B or targeting DYRK1B with the kinase inhibitor AZ191 inhibited liposarcoma cell growth, decreased cell motility, and induced apoptosis. Moreover, combined AZ191 with doxorubicin demonstrated an increased anti-cancer effect on liposarcoma cells. These findings suggest that DYRK1B is critical for the growth of liposarcoma cells. Targeting DYRK1B provides a new rationale for treatment of liposarcoma.

## INTRODUCTION

Liposarcoma is a malignant tumor that arises in the body’s fat cells of the soft tissues. Liposarcoma represents the second most common soft tissue sarcoma and makes up approximately 20% of all sarcomas in adults. This disease occurs most frequently in middle-aged and older adults (age 40 and above), and its predilection sites include the thigh or the retroperitoneum. The classification of soft tissue and bone tumors by the World Health Organization (WHO) in 2013 distinguished four major liposarcoma subtypes: (i) well-differentiated liposarcoma (45% of all liposarcomas); (ii) de-differentiated liposarcoma (5% of all liposarcomas); (iii) myxoid liposarcoma (35% of all liposarcomas); and (iv) pleomorphic liposarcoma (15% of all liposarcomas) [1]. Like other soft tissue sarcomas, a wide resection is the primary standard of treatment for liposarcoma patients, combined with radiotherapy or chemotherapy. Although doxorubicin and ifosfamide have been used for treatment of advanced or metastatic liposarcoma patients for over 30 years, the benefit of chemotherapeutic drugs on survival of metastatic liposarcoma remains controversial [2–4]. The five-year survival rate for patients with high-grade liposarcoma is less than 50% [5]. Therefore, there is an urgent need to identify new treatment strategies to improve the outcomes of patients with liposarcoma.

The human genome contains at least 600 protein kinases that phosphorylate proteins at more than 250,000 sites. Abnormal expression or activation of certain protein kinases involved in tumor cell growth, proliferation, apoptosis, and drug sensitivity [6, 7]. Consequently, protein kinases have emerged as one of the most promising therapeutic target families for the treatment of cancers. To date, the Food and Drug Administration (FDA) has approved over 35 protein kinases inhibitors for clinical use [8]. Several protein kinases have been found to be overexpressed or highly activated in liposarcoma, including the proto-oncogenes phosphatidylinositol 3-kinase (PI3K)/AKT, cyclin-dependent kinase 4 (CDK4), and CDK11 [9–11]. Amplification and upregulation of these kinase pathways are thought to be an early and essential part of the oncogenic program of liposarcoma. For example, CDK4 is amplified in patients with well-differentiated liposarcoma and de-differentiated liposarcoma, hepatocyte growth factor receptor (MET) is overexpressed in de-differentiated liposarcoma, and SRC is activated in myxoid liposarcoma and pleomorphic liposarcoma [12–14]. Recently, clinical trials were conducted to target protein kinases in liposarcoma, including CDK4 inhibitor palbociclib (PD0332991), pan-CDK inhibitor flavopiridol, vascular endothelial growth factor receptor (VEGFR) inhibitor sorafenib, multi-kinase inhibitor regorafenib, and vascular endothelial growth factor A (VEGF-A) monoclonal antibody bevacizumab [15–19].

DYRK1B (also known as Mirk) belongs to the DYRK family of protein kinases, which include five conserved members, DYRK1A, DYRK1B, DYRK2, DYRK3, and DYRK4. DYRKs are dual function kinases family with the ability to auto-phosphorylate themselves on tyrosine during translation, and then phosphorylate other substrates on serine and threonine residues. DYRKs play key roles in the regulation of cell differentiation, proliferation, and survival, as well as cell-cycle control [20]. DYRK1B is mainly highly expressed in skeletal muscle and in the testis and is detected at low levels in most other normal tissues. DYRK1B facilitates skeletal myoblast differentiation and survival by mediating cell cycle arrested in G_0_ phase and exerting anti-apoptotic effect [21, 22]. More importantly, DYRK1B is overexpressed and highly activated in many types of solid tumors, including pancreatic, lung, ovarian, colon cancer, rhabdomyosarcoma and osteosarcoma [23–28]. On the other hand, emerging insights into DYRK1B promotion adipogenesis and involvement in metabolic syndrome suggest that DYRK1B may potentially relevant to fat cell malignancy [29]. Moreover, the roles of DYRK1B in liposarcoma and the significance of targeting DYRK1B signaling as a putative therapeutic remain unknown. Therefore, in the present study, we performed an immunohistochemistry (IHC) assay to examine the expression of DYRK1B in a microarray of liposarcoma patient tissues. We further evaluated the function of DYRK1B in the proliferation and motility of liposarcoma cells. In addition, we determined the effect of the combination of doxorubicin with DYRK1B kinase inhibitor AZ191 on liposarcoma cells.

## RESULTS

### DYRK1B is overexpressed in human liposarcoma as compared with lipoma tissues

**Figure 1:**
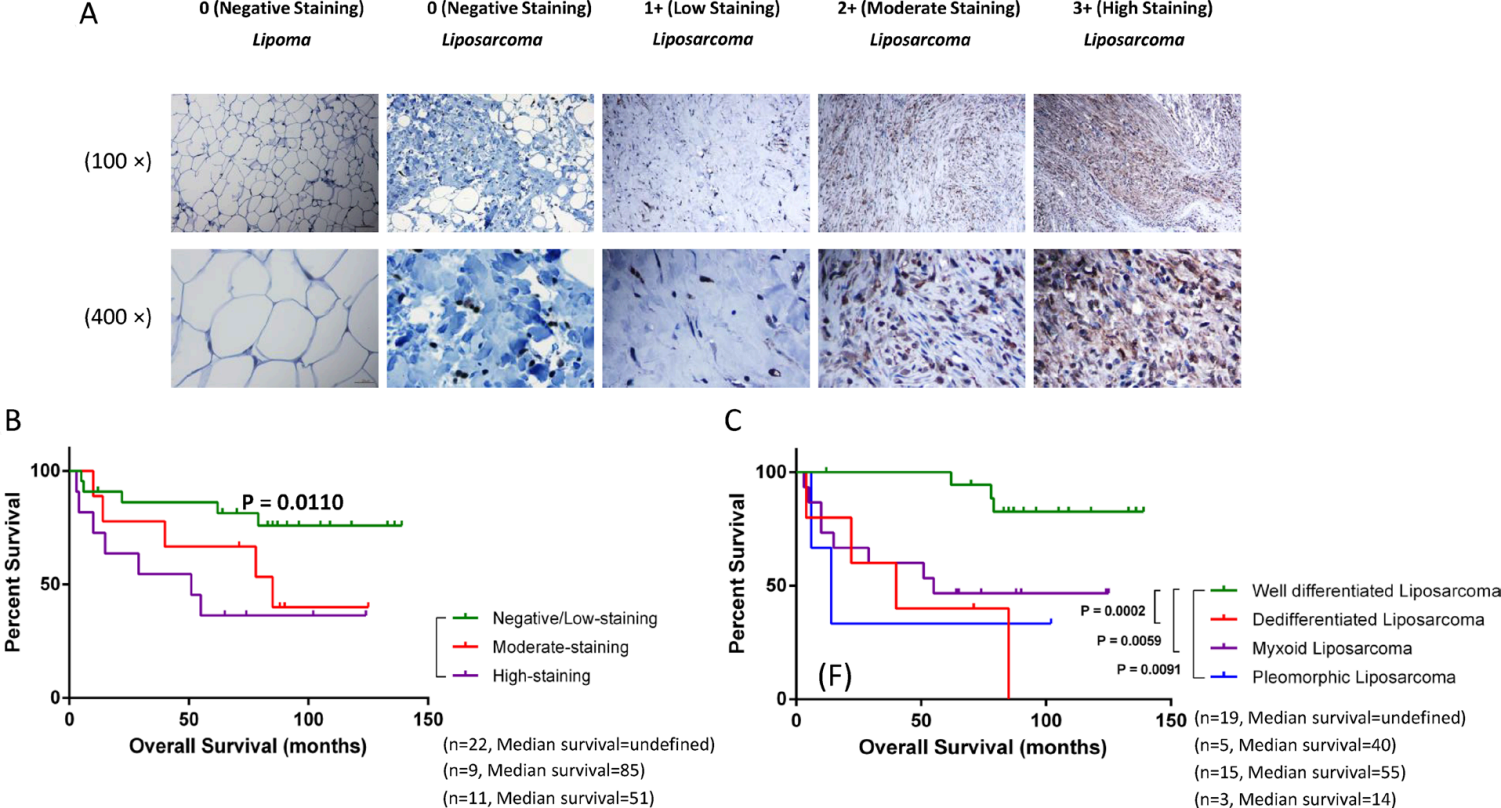
DYRK1B is overexpressed in liposarcoma and correlates with poor patient prognosis (**A**) Representative images of cytoplasmic staining intensity of DYRK1B staining in human lipomatous tumor tissues. Original magnification: 100× and 400×. Scale bar = 100μm (upper 100× photos) or 25 μm (lower 400× photos). (**B**) Correlation between expression of DYRK1B (negative/low staining, moderate staining, and high staining) and overall survival in liposarcoma patients. (**C**) Four liposarcoma pathology subtypes patients (well-differentiated liposarcoma, de-differentiated liposarcoma, myxoid liposarcoma, and pleomorphic liposarcoma) distributed on the Kaplan-Meier survival curve.

We examined the expression of DYRK1B by IHC using a human lipomatous tumor tissue microarray (TMA) and human normal tonsil tissue as control (Figure 1 and Supplementary Figure 1). Seventeen and 42 patients with pathological diagnosis of lipoma and liposarcoma were identified, respectively (Table 1). DYRK1B staining intensity was graded into four groups: 0 (negative staining), 1+ (low staining), 2+ (moderate staining), and 3+ (high staining). The summary of the clinicopathologic characteristics of patients with liposarcoma is shown in Table 2. The results demonstrated that the level of DYRK1B expression were higher in patients with liposarcoma than lipoma patients. In addition, the results also showed that the DYRK1B protein was predominantly localized in the cytoplasm of liposarcoma cells (Figure 1A).

**Table 1:** Characteristics of the lipomatous tumors patients

Factors	No. (%)			
Age				
< 40 years old	9 (15.3%)			
≥ 40 years old	50 (84.7%)			
Gender (*n =* 59)				
Female	26 (44.1%)			
Male	33 (55.9%)			
Pathology (*n =* 59)				
Lipoma	17 (28.8%)			
Liposarcoma, well differentiated	19 (32.2%)			
Liposarcoma, dedifferentiated	5 (8.5%)			
Liposarcoma, myxoid	15 (25.4%)			
Liposarcoma, pleomorphic	3 (5.1%)			
Survival (Liposarcoma *n =* 42)				
Alive	24 (57.1%)			
Deceased	18 (42.9%)			
Tumor Location (Liposarcoma *n =* 42)				
neck/chest/abdomen/buttock	5 (11.9%)			
mediastinum	1 (2.4%)			
abdominal cavity	10 (23.8%)			
retroperitoneal	11 (26.2%)			
Extremities	15 (35.7%)			
Expression of DYRK1B				
	0	1+	2+	3+
Lipoma (*n =* 17)	13 (76.5%)	3 (17.6%)	1 (5.9%)	
Liposarcoma (*n =* 42)	9 (21.4%)	13 (31%)	9 (21.4%)	11 (26.2%)

Note: 0 (negative staining); 1+ (low staining); 2+ (moderate staining); 3+ (high staining).

**Table 2:** Summary of the clinicopathological characteristics of lipomatous tumors patients

Clinicopathological feature	No.	DYRK1B expression			
		Negative/Low staining	Moderate staining	high staining	*p* value
Pathology					
Lipoma	17	16	1	0	0.0089
Liposarcoma	42	22	9	11	
Age (liposarcoma, *n =* 42)					
< 40 years old	7	4	1	2	0.8805
≥ 40 years old	35	18	8	9	
Gender (liposarcoma, *n =* 42)					
Female	21	13	4	4	0.4368
Male	21	9	5	7	
Pathology (liposarcoma, *n =* 42)					
well differentiated	19	18	1	0	0.0001
dedifferentiated	5	1	3	1	
myxoid	15	2	4	9	
pleomorphic	3	1	1	1	

### Expression of DYRK1B is associated with poor prognosis in patients with liposarcoma

To explore the relationship between DYRK1B expression and clinical prognosis, we evaluated DYRK1B staining in liposarcoma patient specimens. Kaplan-Meier survival analysis showed that the prognosis for liposarcoma patients with high DYRK1B staining was significantly worse than those with negative/low DYRK1B staining group (Figure 1B). With respect to clinical characteristics, liposarcomas are classified into four pathology subtypes. Therefore, we compared DYRK1B staining intensity between the four groups: well-differentiated liposarcoma, de-differentiated liposarcoma, myxoid liposarcoma, and pleomorphic liposarcoma (Table 2). Overall survival for patients with well-differentiated liposarcoma was significantly better than the de-differentiated liposarcoma patients, myxoid liposarcoma, and pleomorphic liposarcoma, respectively (Figure 1C).

### DYRK1B is crucial for liposarcoma cell growth and survival

**Figure 2:**
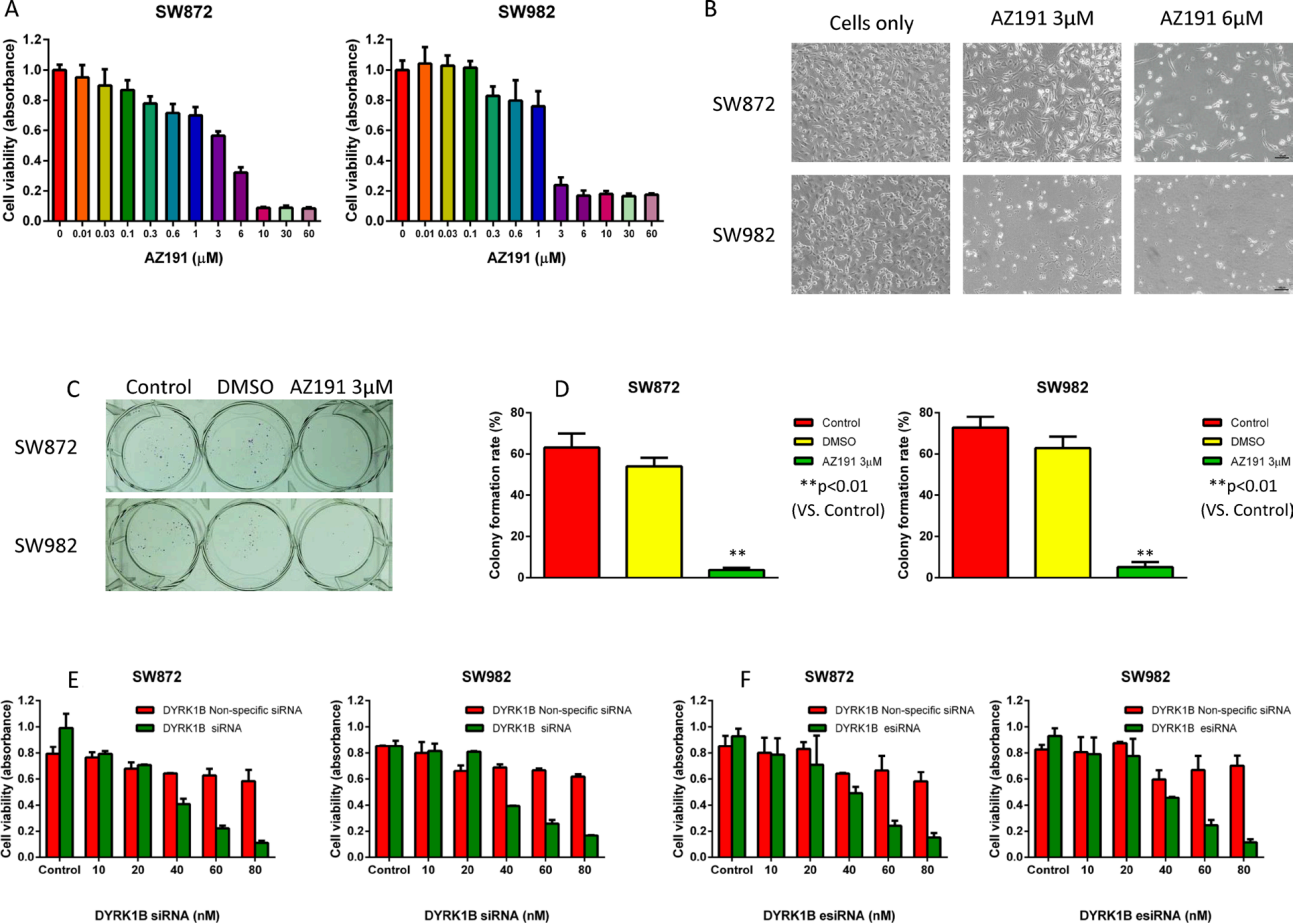
Inhibition of DYRK1B by kinase inhibitor AZ191 or RNAi blocks proliferation in liposarcoma cell lines (**A**) Inhibition of cell proliferation after DYRK1B inhibitor AZ191 treatment of SW872 and SW982 cell lines as determined by the MTT assay. (**B**) Representative images of liposarcoma cells after treatment with increasing concentrations of DYRK1B inhibitor AZ191. Original magnification: 100×. Scale bar = 100 μm. (**C**) Representative images of liposarcoma cell colony formation after treatment with AZ191. (**D**) Inhibition of cell colony formation rate after AZ191 treatment of SW872 and SW982 cells determined by the clonogenic assay. (**E**) Inhibition of cell proliferation after DYRK1B siRNA transfection in SW872 and SW982 cell lines as determined by the MTT assay. (**F**) Inhibition of cell proliferation after DYRK1B esiRNA transfection in SW872 and SW982 cell lines as determined by the MTT assay. Data were shown as means ± S.D.

To characterize the functional role of DYRK1B in liposarcoma, we investigated the inhibition effect of DYRK1B in liposarcoma by small molecule kinase inhibitor AZ191 and RNAi. AZ191 is a novel selective DYRK1B kinase inhibitor [30]. To determine the specific inhibitory effects of DYRK1B on liposarcoma cells *in vitro*, we treated both SW872 and SW982 liposarcoma cell lines with increasing concentrations (0.01–60 μM) of AZ191 for five days and subsequently examined cell viability by 3-(4, 5-dimethylthiazolyl-2)-2, 5-diphenyltetrazolium bromide (MTT) assay. The inhibition of cell viability effects were observed in both cell lines (Figure 2A and 2B). There was dose-dependent growth inhibition with AZ191 treatment in SW872 (IC50 = 3.183 μM) and SW982 (IC50 = 1.279 μM) cell lines. Clonogenicity is a reflection of *in vitro* cell survival based on the ability of a single cancer cell to grow into a colony [31]. To assess the effect of DYRK1B inhibition on survival and proliferation capacity of liposarcoma cells, cells were incubated with 3 μM AZ191 for 1–2 weeks. As shown in Figure 2C and 2D, both SW872 and SW982 cells colony formation capacities were significantly decreased after treatment with AZ191.

To determine the effect of DYRK1B knockdown on liposarcoma cell growth *in vitro*, we transfected synthetic DYRK1B siRNA into SW872 and SW982 cell lines. After incubation with DYRK1B siRNA or non-specific siRNA at concentrations ranging from 10 to 80 nM for five days, the MTT assay showed that siRNA-mediated knockdown of DYRK1B significantly reduced liposarcoma cell viability in both cell lines in a dose-dependent manner (Figure 2E).

**Figure 3:**
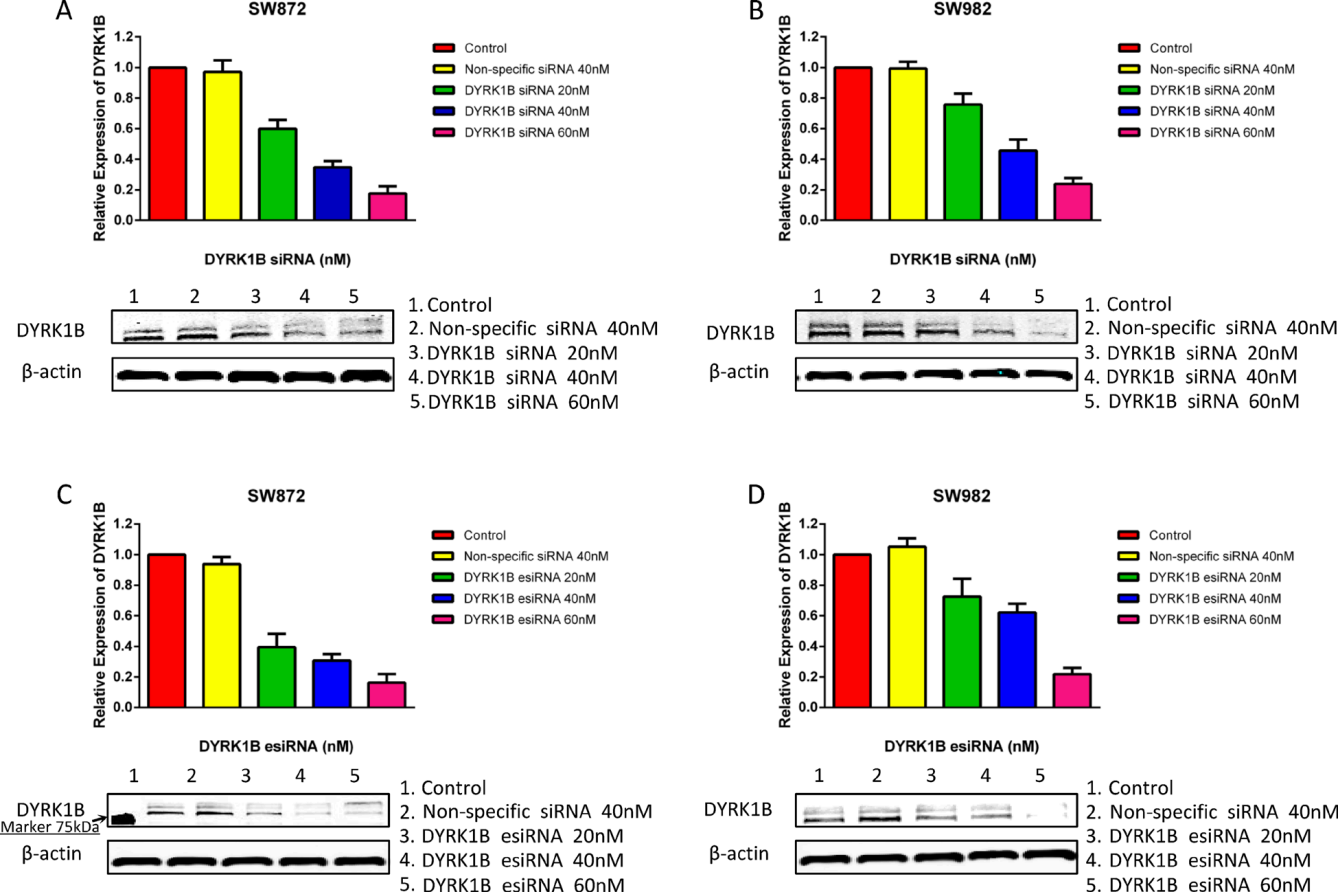
DYRK1B protein expression after treatment of liposarcoma cell lines with DYRK1B siRNA or esiRNA as determined by Western blot (**A** and **B**) Knockdown of DYRK1B protein expression after DYRK1B siRNA transfection in SW872 and SW982 cell lines as determined by Western blot and semi-quantitative analysis. (**C** and **D**) Confirmation of knockdown of DYRK1B protein expression after DYRK1B esiRNA transfection in SW872 and SW982 cell lines as determined by Western blot and semi-quantitative analysis. Molecular size marker 75kDa is shown at the left.

To further confirm the effect of DYRK1B knockdown on liposarcoma cells, we also applied DYRK1B targeted endoribonuclease-prepared siRNA (esiRNA) in liposarcoma cell lines. esiRNAs are synthesized by *in vitro* transcription of a 300–600 bp gene specific double-stranded RNA (dsRNA), followed by enzymatic digestion using RNases (i.e., RNase III). esiRNAs have the advantage of lower off-target effects as compared with siRNA [32]. Similar results to the siRNA treatment were obtained with DYRK1B esiRNA (concentration gradient ranged from 10 to 80 nM for five days) in both SW872 and SW982 cell lines (Figure 2F). Downregulated expression of DYRK1B was confirmed by Western blot after DYRK1B siRNA or esiRNA transfection for 48 hours (Figure 3A–3D). In addition, two DYRK1B blot bands were detected. The molecular weight was around 70 kDa and 67 kDa, in line with previous studies [33, 34]. The level of DYRK1B expression was suppressed by DYRK1B targeted RNAi in a dose-dependent manner, as determined by densitometry quantification. These results demonstrate that DYRK1B is vital to promote cell growth and viability in liposarcoma cells.

### Inhibition of DYRK1B suppresses liposarcoma cell motility

**Figure 4:**
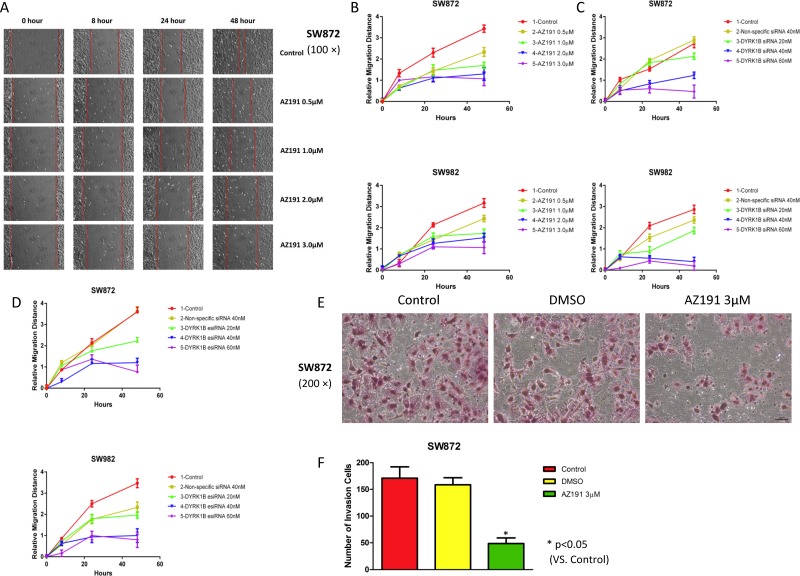
Inhibition of DYRK1B by kinase inhibitor AZ191 or RNAi impairs motility in liposarcoma cell lines (**A**) Representative migration images of SW872 cell lines at different time points (0, 8, 24, and 48 hours) when treated with different concentrations of AZ191. Original magnification: 100×. Scale bar = 100 μm. (**B**) Relative migration distance of SW872 and SW982 at different time points (0, 8, 24, and 48 hours) when treated with different concentrations of AZ191. (**C**) Relative migration distance of SW872 and SW982 at different time points (0, 8, 24, and 48 hours) when treated with different concentrations of DYRK1B siRNA and non-specific siRNA. (**D**) Confirmation of relative migration distance of SW872 and SW982 at different time points (0, 8, 24, and 48 hours) when treated with different concentrations of DYRK1B esiRNA and non-specific siRNA. (**E** and **F**) Representative invasion images and number of invasion SW872 cells through the Matrigel after treatment with DMSO or AZ191 3 μM for 48 hours. Original magnification: 200×. Scale bar = 50 μm. Data were shown as means ± S.D.

Tumor cell motility is important for cancer invasion and metastasis. We next investigated the effect of DYRK1B inhibition on the abilities of liposarcoma cell migration and invasion. Wound healing assays were performed after treatment with AZ191, DYRK1B siRNA, or esiRNA. Relative cell migration distance was evaluated at 0, 8, 24, and 48 hours, respectively, after treatment by the scratch assay as described. We observed a marked inhibition of migratory potential in both SW872 and SW982 cell lines compared with control or non-specific siRNA groups, especially at higher concentrations groups (Figure 4A–4D and Supplementary Figure 2A–2C). Wounds were almost fully recovered after the 48-hour migration in blank control and non-specific siRNA treated cells. Similarly, in transwell invasion chamber assays, SW872 cell invasion ability was significantly reduced compare with control cells after treatment with AZ191 3 μM for 48 hours (*P* < 0.05, Figure 4E and 4F). These data demonstrate that inhibition of DYRK1B impaired liposarcoma cell motility capability.

### DYRK1B inhibition induces apoptosis in liposarcoma cells

**Figure 5:**
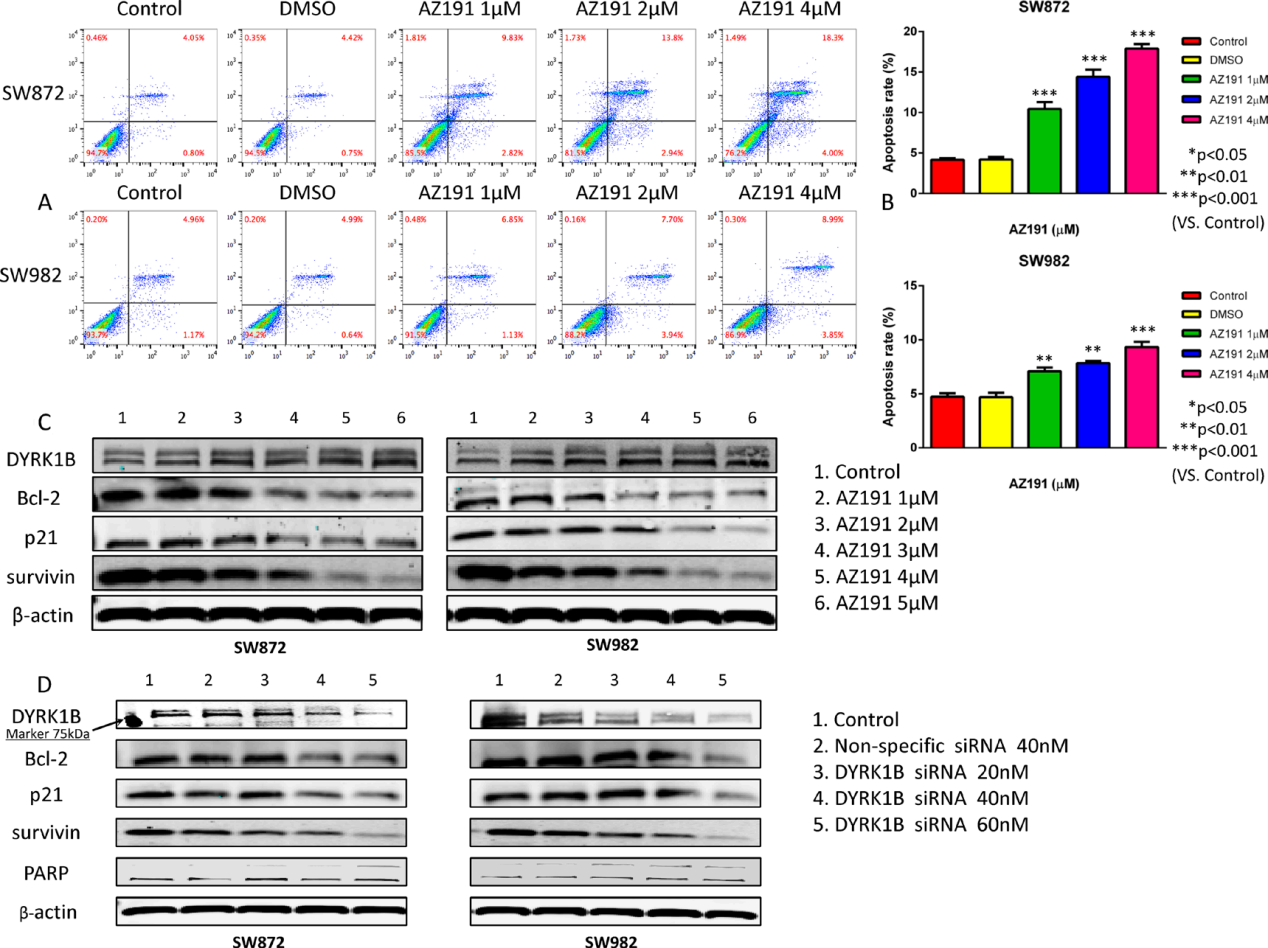
Inhibition of DYRK1B by kinase inhibitor AZ191 or siRNA transfection induces cell apoptosis in liposarcoma cell lines (**A**) Representative results of flow cytometric analysis cell apoptosis of SW872 and SW982 cell lines, incubated with different concentrations of AZ191 or DMSO. (**B**) Analysis of apoptosis rate of SW872 and SW982 cell lines treated with different concentrations of AZ191 or DMSO. (**C**) Representative Western blot analysis of apoptosis-related proteins alterations in SW872 and SW982 cell lines after treatment with different concentrations of AZ191. (**D**) Representative Western blot analysis of apoptosis-related proteins alterations in SW872 and SW982 cell lines after transfection of different concentrations of DYRK1B siRNA and non-specific siRNA. Molecular size marker 75kDa is shown at the left. Data were shown as means ± S.D.

To assess whether the observed growth inhibitory effect induced by DYRK1B inhibition on liposarcoma cells was due to an induction of apoptosis, we performed flow cytometric analysis and Western blot assays. Following 48 hours of treatment with 1 μM, 2 μM, and 4 μM AZ191 as described, both liposarcoma cell lines showed significantly increased rates of apoptosis in a dose-dependent fashion as compared with dimethyl sulfoxide (DMSO) treated cells, which were used as negative controls (Figure 5A and 5B).

Then, we conducted Western blot assays on cell extracts by treatment with increasing concentrations of AZ191 or DYRK1B siRNA after 48 hours. The Western blot assay showed that targeting DYRK1B with AZ191 did not alter the expression of DYRK1B protein levels (Figure 5C), which is consistent with previous literature [30]. Several apoptotic related proteins were assessed. As shown in Figure 5C, downregulation of three anti-apoptotic proteins (Bcl-2, p21, and survivin) were observed at higher concentrations of AZ191 treated cells as compared with controls. Similar results were obtained in DYRK1B siRNA transfected liposarcoma cell lines (Figure 5D).

### DYRK1B inhibition increases anti-cancer effects of doxorubicin

**Figure 6:**
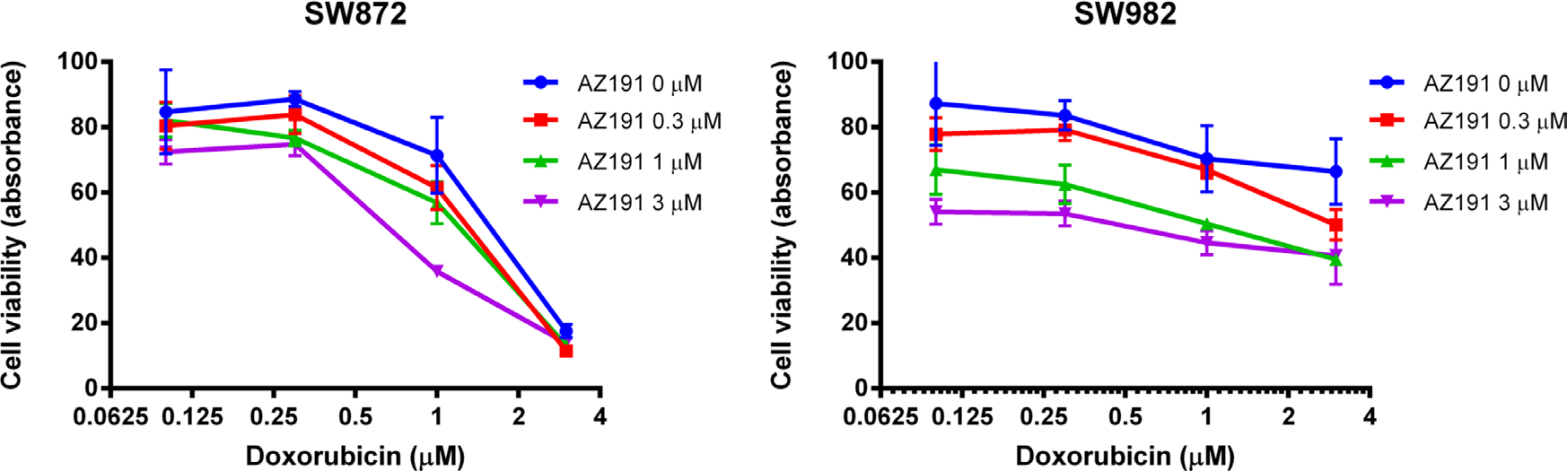
Co-incubation of different concentrations of AZ191 with increasing concentrations of doxorubicin increased anti-cancer effects in SW872 and SW982 cell lines as determined by the MTT assay

To determine the effects of combinations of conventional chemotherapy agent doxorubicin and DYRK1B targeted therapy on the growth of liposarcoma cells, both SW872 and SW982 cells were co-treated with increasing doses of doxorubicin and AZ191 for five days. As shown by MTT analysis, the combination of doxorubicin with AZ191 increased anti-cancer effects on both liposarcoma cell lines (Figure 6).

## DISCUSSION

In the present study, we demonstrated for the first time the crucial role of DYRK1B in liposarcoma. It has been previously demonstrated that DYRK1B was classified into DYRK1B-p65, DYRK1B-p69 and DYRK1B-p75 three splicing variants subtypes with different expression patterns and protein kinases activities [33]. Moreover, it is proposed that DYRK1B-p65 and DYRK1B-p69 quite probably correspond to DYRK1B 70 kDa and 67 kDa described by earlier report [34]. More importantly, the previous study described that undifferentiated 3T3-L1 preadipocytes contained only DYRK1B-p65 and DYRK1B-p69. This result seems likely that there may be a relationship between DYRK1B and liposarcoma since liposarcoma is considered a malignant tumor arises in the fat cells. Our findings demonstrated the DYRK1B protein is overexpressed in the majority of liposarcoma patient specimens as compared with lipoma tissues by IHC analysis. We then examined the association between the level of DYRK1B expression and the prognostic significance of pathology subtype in liposarcomas. We also demonstrated that higher expression of DYRK1B is correlated with worse prognosis in liposarcoma. Kaplan-Meier survival curve analysis showed that well-differentiated liposarcoma patients have a better prognosis than other pathology subtypes [35]. These findings validate previous reports that amplified expression of DYRK1B is involved in the progression of certain cancers and associated with poor prognosis [36–40]. We then investigated the function roles of DYRK1B in liposarcoma cells. By targeting with small molecule kinase inhibitor AZ191 or RNAi-mediated knockdown, we observed reduction of proliferation, as well as suppression of cell motility, induction of apoptosis, and sensitization to chemotherapy drug in liposarcoma cells. These findings indicate that DYRK1B could play a significant role in liposarcoma cell growth and proliferation.

We then investigated the potential mechanism of inhibition of DYRK1B on the growth of liposarcoma cells. Several small molecule DYRK1B kinase inhibitors have been studied in preclinical cancer researches, including DYRKi in pancreatic cancer, and EHT5372 in pancreatic cancer and ovarian cancer [40–42]. We recruited DYRK1B kinase inhibitor AZ191 to block DYRK1B kinases activity, which has been validated in pancreatic and ovarian cancer cells before [43]. AZ191 has been shown to exhibit 10-fold selectivity for DYRK1B over DYRK1A [30]. As demonstrated by flow cytometric analysis, AZ191 induced apoptosis in both liposarcoma cell lines in a dose-dependent manner. Expression of anti-apoptotic proteins, including Bcl-2, p21, and survivin, were decreased after treatment with AZ191, suggesting that DYRK1B is involved in apoptosis signaling. Bcl-2 is the founding member of the Bcl-2 family—a well-established key regulator of the apoptosis gene family. Bcl-2 exerts a cell survival function in response to apoptotic stimuli through inhibition of mitochondrial cytochrome C release, which subsequently activates caspase and Poly(ADP-ribose) polymerase (PARP) cleavage in the intrinsic apoptosis pathway [44]. Our finding was consistent with a previous report describing that downregulation of Bcl-2 was involved in apoptosis in SW872 cells [45]. Survivin belongs to the inhibitor of apoptosis (IAP) family and has anti-apoptosis function by directly binding and inhibiting caspase-3 activation, which serves as a critical effector of apoptosis [46]. Elevated survivin also was reported in soft tissue sarcoma, including liposarcoma, and inverse correlated with prognosis [47]. Depending on intracellular localization, p21 plays a role in cell cycle arrest as a potent inhibitor of apoptosis, which is also involved in p53 mediated apoptosis and interaction with procaspase-3 and cyclin A/CDK2 complexes [48, 49]. More interestingly, DYRK1B can phosphorylate p21 at Ser153 and drive p21 translocation to the cytoplasm, which subsequently enables p21 to block apoptosis [22, 50]. In addition, DYRK1B also promotes the expression of p21 [30]. In line with these findings, we observed that AZ191 reduced the expression of p21 in liposarcoma cells associated with increased apoptosis.

**Figure 7:**
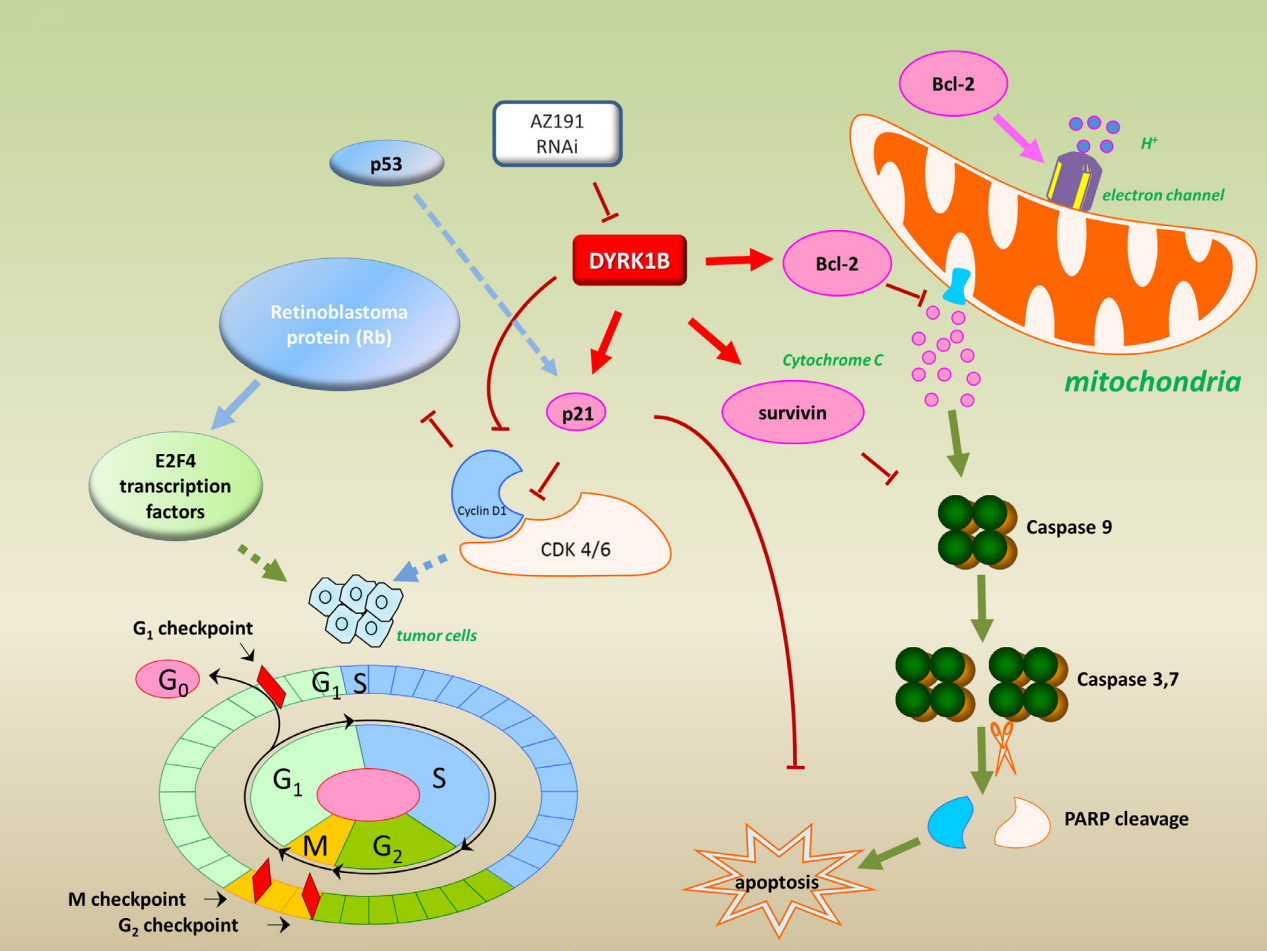
Model for targeting DYRK1B to regulate cell proliferation and apoptosis in liposarcoma Inhibition of DYRK1B by kinase inhibitor AZ191 or RNAi down-regulation of anti-apoptotic proteins (Bcl-2, p21, and survivin) were observed and are represented.

To further confirm the effects of inhibition DYRK1B in liposarcoma, we compared transfection with DYRK1B siRNA to SW872 and SW982 liposarcoma cell lines. Knockdown of DYRK1B by siRNA also resulted in decreased Bcl-2, p21, and survivin. Moreover, PARP cleavage was detected by knockdown of DYRK1B. These data further validate the molecular mechanism for transfection of DYRK1B siRNA induced apoptosis in liposarcoma. Taken together, as shown in Figure 7, our study suggests that inhibition of DYRK1B with RNAi or a specific kinase inhibitor AZ191 suppresses cell proliferation and induces apoptosis through the downregualtion of anti-apoptotic proteins in liposarcoma.

Finally, we found that inhibition of DYRK1B with AZ191 enhanced the cytotoxic effect of doxorubicin in liposarcoma cells, which is consistent with previous reports that DYRK1B inhibitor sensitized both ovarian cancer cell lines and patient ascites derived primary cells to chemotherapy drug cisplatin [42, 51]. This data suggests that a combination therapy of DYRK1B inhibition and chemotherapy drug could be considered for clinical trials as a potent treatment for liposarcoma patients.

In summary, our study revealed that DYRK1B is overexpressed in liposarcoma. High expression of DYRK1B is associated with poor outcomes, which may serve as a prognostic and predictive biomarker in liposarcoma patients. Inhibition of DYRK1B resulted in significantly decreased cell growth and motility in liposarcoma. This effect was enhanced when combined with doxorubicin. Future *in vivo* studies and molecular mechanisms of DYRK1B in liposarcoma should be explored.

## MATERIALS AND METHODS

### Tissue Microarray (TMA) and immunohistochemistry (IHC)

The conditions of DYRK1B IHC were first evaluated and optimized in human normal tonsil tissues with the rabbit polyclonal antibody to human DYRK1B, which was kindly provided by Dr Xiaobing Deng and Ying Huang from State University of New York, Syracuse, New York. Normal tonsil showed low expression of DYRK1B (Supplementary Figure 1). The human liposarcoma and lipoma tissue array was purchased from Novus Biologicals, LLC (Littleton, CO, USA) and were used in accordance with the policies of the institutional review board of the Massachusetts General Hospital (IRB protocol # 2007P-002464), which contains 59 samples from 59 lipomatous tumors patients, and included clinicopathological data on age, sex, location, diagnosis, tumor tissue pathological subtypes, follow-up months, follow-up result, and cause of death. The expression level of DYRK1B was determined based on the Immunohistochemistry Protocol (Paraffin) from Cell Signaling Technology (Danvers, MA, USA). Briefly, paraffin embedded slide was baked at 60°C for 1 hour. Sections were washed three times in xylene for 5 min each, and then transferred through graded ethanol (100% and 95%) twice for rehydration for 10 minutes each. Following the process of antigen retrieval, endogenous peroxidase activity was quenched by incubation in 3% hydrogen peroxide. After protein blocking with normal goat serum for 1 hour at room temperature, the DYRK1B primary antibody (1:50 dilution) was applied at 4°C overnight in a humidified chamber. The bound antibody on the array was detected by using SignalStain^®^ Boost Detection Reagent (Cell Signaling Technology, Danvers, MA, USA) and SignalStain^®^ DAB (Cell Signaling Technology, Danvers, MA, USA). Prior to imaging, the section was counterstained with hematoxylin QS (Vector Laboratories, CA, USA) and mounted with VectaMount AQ (Vector Laboratories, CA, USA) coverslip for long-term preservation.

The degree of immunostaining on the tissue array was viewed and scored separately by two independent investigators who had no knowledge of the histopathological features or patient details of the samples. Any differences in the scores were resolved by consensus after joint review of the slides and discussion between the two investigators. The immunostaining intensity pattern of DYRK1B was assessed on a scale semi-quantitatively as follows: 0, negative staining; 1+, low staining; 2+, moderate staining; and 3+, high staining.

### Cell lines and cell culture

The human liposarcoma cell lines SW872 and SW982 were purchased from the American Type Culture Collection (Rockville, MD, USA) in 2013 with certificate of analysis. Both the liposarcoma cell lines were cultured in RPMI-1640 medium (Life Technologies, Grand Island, NY, USA) supplemented with 10% fetal bovine serum (Sigma-Aldrich, St. Louis, MO, USA), penicillin (100 mg/ml), and streptomycin (100 mg/ml; Invitrogen, Grand Island, NY, USA). All cells were maintained in a humidified incubator containing 5% CO_2_–95% air atmosphere at 37°C.

### Cell proliferation assay and clonogenic assay

Cells were exposed to various treatments (DYRK1B inhibitor, siRNA, esiRNA, or vehicle control) for respective time points as indicated. Cell proliferation ability and cytotoxicity of inhibiting DYRK1B were assessed by 3-(4, 5-dimethylthiazolyl-2)-2, 5-diphenyltetrazolium bromide (MTT) assays. In brief, liposarcoma cells were seeded at 3 × 10^3^ cells per well into 96-well plates with complete growth medium without antibiotics in triplicate and exposed to increasing concentrations (0.01–60 μM) of small molecule DYRK1B inhibitor AZ191 (Selleck Chemicals, Houston, TX, USA) for five days. Subsequently, 20 μl MTT (Sigma-Aldrich, St. Louis, MO, USA) was added to each well and then incubated for 4 hours at 37°C and 5% CO_2_ humidified atmosphere. Then, the MTT formazan product was dissolved with acid isopropanol. The absorbance was assessed on a SpectraMax Microplate^®^ Spectrophotometer (Molecular Devices LLC, Sunnyvale, CA, USA) at 490 nm. Liposarcoma cells were seeded at 3 × 10^3^ cells per well into 96-well plates in duplicate, and treatment with human non-specific siRNA (MISSION^®^ siRNA Universal Negative Control, SIC001, Sigma-Aldrich, St. Louis, MO, USA) and synthetic human DYRK1B siRNA (Genebank Accession Number:NM_004714, coding regions sense 5′-GGCACUUCAUGUUCCGGAAtt-3′, antisense 5′-UUCCGGAACAUGAAGUGCCgc-3′, SIHK0641, Sigma-Aldrich, St. Louis, MO, USA) or esiRNA human DYRK1B (EHU076811, Sigma-Aldrich, St. Louis, MO, USA) at concentrations ranging from 10 to 80 nM for five days. Cells were imaged on a Nikon Eclipse Ti-U microscope (Nikon Corp., Melville, New York, USA) equipped with a SPOT RT™ digital camera from Diagnostic Instruments, Inc. (MI, USA). For clonogenic assay, SW872 and SW982 cells were seeded at 3 × 10^2^ cells per well into 6-well plates, incubated with AZ191 for 1–2 weeks, then methanol fixed and Giemsa (GS, Sigma-Aldrich, St. Louis, MO, USA) stained, followed by colony counting.

### Knockdown of DYRK1B by RNA interference

DYRK1B knockdown in human liposarcoma cells was performed by siRNA or esiRNA transfection. The non-specific siRNA oligonucleotides were used as negative controls. 2 × 10^5^ liposarcoma cells per well were seeded in 12-well plates with complete growth medium without antibiotics. Various concentrations (0, 20, 40, and 60 nM) of DYRK1B siRNA/esiRNA or non-specific siRNA (40nM) were transfected into cells using Lipofectamine RNAiMax Reagent (Invitrogen, CA, USA) according to the manufacturer’s instructions. After 48 hours, transfected cells were subjected to subsequent Western blot analysis.

### Protein preparing and western blotting

Protein lysates of the cells were extracted with 1× RIPA lysis buffer (Upstate Biotechnology, Charlottesville, VA, USA) supplemented with complete protease inhibitor cocktail tablets (Roche Applied Science, IN, USA) after incubation with AZ191 or DYRK1B siRNA/esiRNA for 48 hours. The concentrations of the protein were determined by protein assay reagents (Sigma-Aldrich, St. Louis, MO, USA) with a spectrophotometer (Beckman Du-640, Beckman Instruments, Inc., Indianapolis, IN, USA). Western blotting was performed as follows: denatured proteins were run on NuPAGE^®^ 4–12% Bis-Tris Gel (Life Technologies, Grand Island, NY, USA), and then transferred to nitrocellulose membrane (Bio-Rad, CA, USA). Membranes were blocked in 5% nonfat milk for 1 hour, and incubated with specific primary antibody (rabbit polyclonal antibody to human DYRK1B) or mouse monoclonal antibody to human β-actin (Sigma-Aldrich, St. Louis, MO, USA) at 4°C overnight. Following primary antibody incubation, membranes were washed with PBST (1×), and goat anti-rabbit IRDye^®^ 800CW or goat anti-mouse IRDye^®^ 680LT secondary antibody (1:20000 dilution) (926–32211 and 926–68020, Li-COR Biosciences, NE, USA) was added, respectively. Bands were detected using Odyssey for Infrared Fluorescent Western Blots from Li-COR Bioscience (Lincoln, NE, USA). Quantification analysis of Western blot bands was performed with ImageJ software (National Institutes of Health, USA). All other antibodies used in this study were purchased from Cell Signaling Technology (Danvers, MA, USA).

### Wound healing assay and cell invasion assay

Cell migration activity was evaluated by wound healing assay. In brief, 2 × 10^5^ cells were seeded onto 12-well plates and treated with different concentration of AZ191, DYRK1B siRNA/esiRNA, or non-specific siRNA. After the cells reached 100% confluence, they were wounded by scraping three parallel lines with a 200 µl tip, and then washed three times in serum-free medium and incubated in regular medium. Wounds were observed at 0, 8, 24, and 48 hours, respectively. Three images were taken per well at each time point using a Nikon microscope (10× objective) to monitor the cell repair process, and the distance between the two edges of the scratch (wound width) was measured at three random sites in each image. The cell migration distance was calculated by subtracting the wound width at each time point from the wound width at the 0 hour time point. Transwell invasion chamber assay provided an *in vitro* system to study cell invasion activity with a BD BioCoat™ Matrigel™ Invasion Chamber (Becton-Dickinson, MA, USA). In brief, cell suspensions were prepared containing 5 × 10^4^ cells/well in the upper chambers of 24 well invasion chambers with serum-free medium, while the bottom chambers were filled with 750 μl medium with 10% FBS without antibiotics. After treatment with DMSO or AZ191 3μM for 48 hours, the non-invading cells were carefully scrubbed from the upper surface of the membrane with a cotton swab. Cells were fixed using 100% methanol, stained in hematoxylin for 15 min, and rinsed twice in distilled water. The number of invading cells was counted in three images per membrane under a microscope using a 20× objective.

### Analysis of cell apoptosis by flow cytometry

Cells were exposed to AZ191 or 0.1% DMSO for 48 hours and harvested following the manufacturer’s protocol. Cells were washed twice with cold PBS and then resuspended in 1× Binding Buffer (BD Biosciences, San Diego, CA, USA) at a concentration of 1 × 10^6^ cells/ml. 100 µl of the solution (1 × 10^5^ cells) was transferred to a 5 ml culture tube, and 5 µl of FITC Annexin V (BD Biosciences, San Diego, CA, USA) was added. Then, 10 µl Propidium Iodide (PI) (BD Biosciences, San Diego, CA, USA) was added, the cells were vortexed, and then subsequently incubated for 15 min at room temperature (25°C) in the dark. 400 µl of 1× Binding Buffer was added to each tube, and the cells were analyzed by flow cytometry (BD FACSCalibur, BD Biosciences, San Diego, CA, USA).

### AZ191 in combination with chemotherapy in liposarcoma cell lines

The combination of AZ191 with chemotherapeutic drug doxorubicin to liposarcoma cells were assessed by MTT assay. SW872 and SW982 cells were seeded into 96-well plates at a density of 3×10^3^ cells per well in triplicate and incubated with a series of concentrations of AZ191 and doxorubicin for five days, which was supplied by the pharmacy at the Massachusetts General Hospital. After five days of co-incubation, cell proliferation was determined by the MTT assay as previously described.

### Statistical analysis

The data were analyzed using Prism 5.0 software (Graph Pad Software Inc., San Diego, CA, USA). Statistical significance was assessed using Mann-Whitney test for independent data. One-way ANOVA test was performed for multiple comparisons. Associations between results of IHC and clinicopathological factors were assessed by Chi-square test. Survival analysis was performed using Kaplan-Meier survival curves with Log-rank test for significance. Differences of *P* < 0.05 were considered significant for all statistical tests.

## SUPPLEMENTARY MATERIALS FIGURES



## Abbreviations

DYRK1B: dual-specificity tyrosine-(Y)-phosphorylation regulated kinase 1B
IHC: immunohistochemistry
RNAi: RNA interference
WHO: World Health Organization
FDA: Food and Drug Administration
PI3K: proto-oncogenes phosphatidylinositol 3-kinase
CDK4: cyclin-dependent kinase 4
MET: hepatocyte growth factor receptor
VEGFR: vascular endothelial growth factor receptor
VEGF-A: vascular endothelial growth factor A
esiRNA: endoribonuclease-prepared siRNA
MTT: 3-(4, 5-dimethylthiazolyl-2)-2, 5-diphenyltetrazolium bromide
DMSO: dimethyl sulfoxide
PI: Propidium Iodide
IAP: inhibitor of apoptosis
PARP: Poly(ADP-ribose) polymerase
