# Supplementary material for: Targeting DYRK1B suppresses the proliferation and migration of liposarcoma cells
Source: Oncotarget. 2017 Nov 28;9(17):13154–66. doi: 10.18632/oncotarget.22743 (PMC5862568; doi:10.18632/oncotarget.22743)
Supplement: Supplementary file 1 [file oncotarget-09-13154-s001.pdf]

Targeting DYRK1B suppresses the proliferation and migration of liposarcoma cells

SUPPLEMENTARY MATERIALS

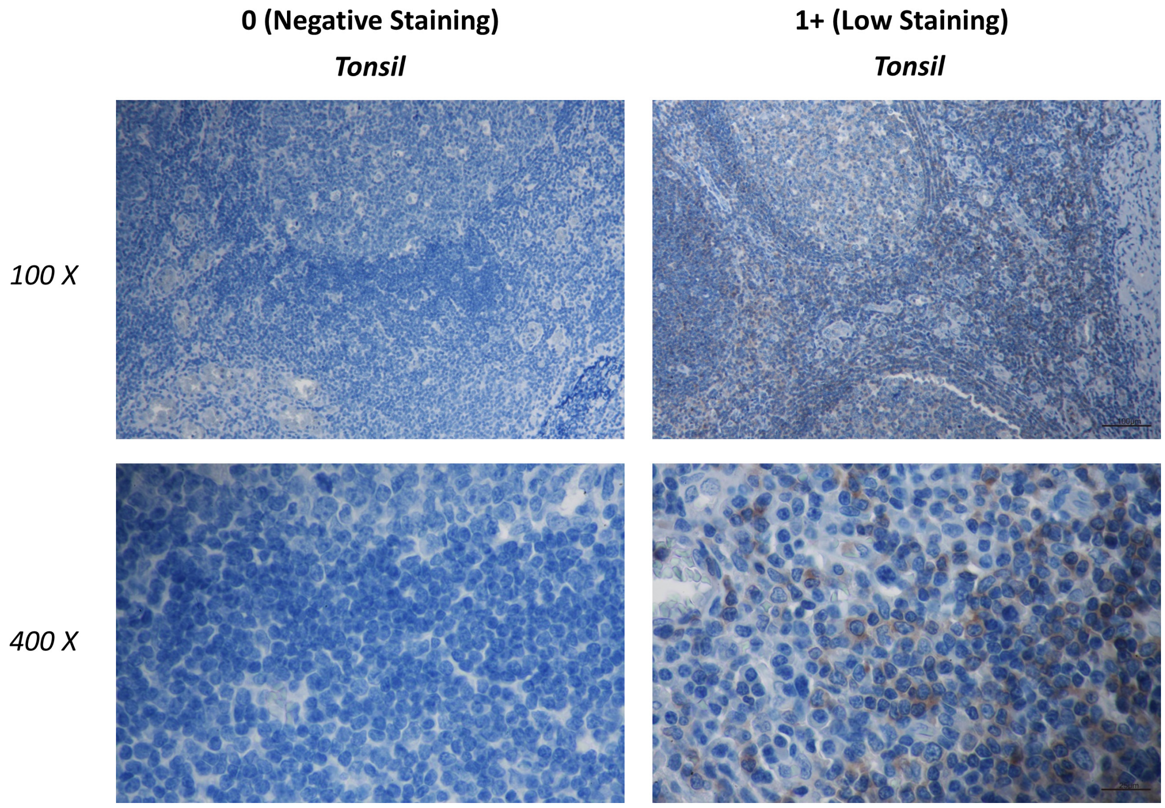

Supplementary Figure 1: Representative images of DYRK1B staining in human normal tonsil tissues as negative control and positive control for IHC. Original magnification: 100× and 400×. Scale bar = 100 µm (upper 100× photos) or 25 µm (lower 400× photos).

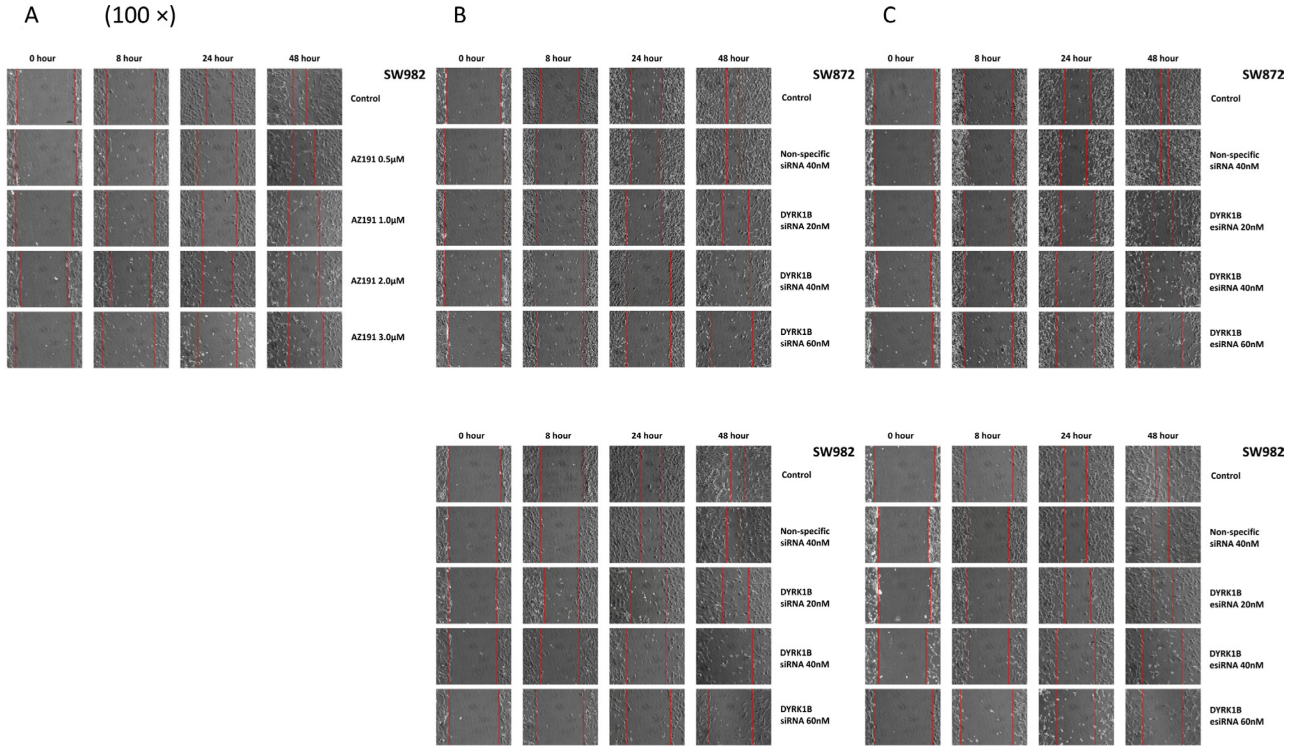

Supplementary Figure 2: Inhibition of DYRK1B by kinase inhibitor or RNAi impairs migration in liposarcoma cell lines. (**A**) Representative migration images SW982 cell lines at different time points (0, 8, 24, and 48 hours) when treated with different concentrations of AZ191. (**B**) Representative migration images of SW872 and SW982 cell lines at different time points (0, 8, 24, and 48 hours) when treated with different concentrations of DYRK1B siRNA and non-specific siRNA. (**C**) Representative migration images of SW872 and SW982 cell lines at different time points (0, 8, 24, and 48 hours) when treated with different concentrations of DYRK1B esiRNA and non-specific siRNA. Original magnification: 100×. Scale bar = 100 μm.
